# Could adaptive deep brain stimulation treat freezing of gait in Parkinson’s disease?

**DOI:** 10.1007/s00415-025-13000-8

**Published:** 2025-03-12

**Authors:** Philipp Klocke, Moritz A. Loeffler, Simon J. G. Lewis, Alireza Gharabaghi, Daniel Weiss

**Affiliations:** 1 Centre for Neurology, Department of Neurodegenerative Diseases, and Hertie Institute for Clinical Brain Research, University of Tübingen, Hoppe-Seyler-Str. 3, 72076 Tübingen, Germany; 2 Parkinson’s Disease Research Clinic, Macquarie Medical School, Macquarie University, 75 Talavera Road, Sydney, NSW Australia; 3 Institute for Neuromodulation and Neurotechnology, University Hospital and University of Tübingen, 72076 Tübingen, Germany; 4 Centre for Bionic Intelligence Tübingen Stuttgart (BITS), University Hospital and University of Tübingen, 72076 Tübingen, Germany; 5 German Centre for Mental Health (DZPG), University Hospital and University of Tübingen, 72076 Tübingen, Germany

**Keywords:** aDBS, Freezing, Parkinson’s disease, Biomarker, Closed-loop

## Abstract

Next-generation neurostimulators capable of running closed-loop adaptive deep brain stimulation (aDBS) are about to enter the clinical landscape for the treatment of Parkinson’s disease. Already promising results using aDBS have been achieved for symptoms such as bradykinesia, rigidity and motor fluctuations. However, the heterogeneity of freezing of gait (FoG) with its wide range of clinical presentations and its exacerbation with cognitive and emotional load make it more difficult to predict and treat. Currently, a successful aDBS strategy to ameliorate FoG lacks a robust oscillatory biomarker. Furthermore, the technical implementation of suppressing an upcoming FoG episode in real-time represents a significant technical challenge. This review describes the neurophysiological signals underpinning FoG and explains how aDBS is currently being implemented. Furthermore, we offer a discussion addressing both theoretical and practical areas that will need to be resolved if we are going to be able to unlock the full potential of aDBS to treat FoG.

## Introduction

Freezing of gait (FoG) affects 50–80% of patients with idiopathic Parkinson’s disease (PD) [1] and leads to significant morbidity, impairments in quality of life, and an increased need for nursing home care as the disease progresses [2–5]. In addition, FoG is a common symptom in other Parkinsonian syndromes, including multiple system atrophy, progressive supranuclear palsy, vascular encephalopathy, and normal pressure hydrocephalus [6], where even less is known about its pathophysiology. Previously, FoG has been defined as a ‘brief episodic absence or marked reduction of forward progression of the feet despite the intention to walk’[7]. However, it has been recognized that more specific definitions and standardized assessments are needed to address clinical and research applications, which are presently being addressed by the International Consortium for Freezing of Gait (IC-FoG) [8, 9].

The neuronal circuit mechanisms underlying FoG remain poorly explored leaving present neuromodulation approaches only partly effective [10]. To date, neurostimulation approaches to interfere with FoG using deep brain stimulation (DBS) have been reported for the subthalamic nucleus (STN) [11, 12], pedunculopontine nucleus (PPN) [13, 14], substantia nigra pars reticulata (SNr) [15], as well as the spinal cord[16]. Several studies have demonstrated reproducible efficacy of STN-DBS on levodopa-sensitive FoG [11, 17–23]. However, the long-term benefits of STN-DBS are more questionable as levodopa-resistant symptoms of the late disease stage emerge with axial motor impairments [24–31], alongside contributions from cholinergic and noradrenergic dysfunction [32, 33]. Continuous (open-loop) delivery of conventional STN-DBS (cDBS) neither accounts for the episodic occurrence of FoG, nor the dynamic nature of gait [34–36]. As such, the field is presently undergoing a period of transformation, moving away from chronic cDBS to an adaptive (closed-loop) DBS (aDBS), with anticipations to specifically target pathological neural dynamics.

Over the past 10 years, a few aDBS studies have led to greater improvement in bradykinesia-rigidity compared to cDBS [37–39]. In addition, other studies have reported non-inferior outcomes with higher stimulation efficiency and lower rates of stimulation-induced side effects [40–45]. However, very few aDBS studies have specifically focused on gait impairment and FoG with substantial variations in its implementation and outcome parameters [46–48]. Furthermore, methodological and technical barriers directly hamper the effective implementation of aDBS for FoG [49]. Thus, while aDBS offers promise for the treatment of FoG, its benefits are not yet clear. Therefore, this targeted review aims to summarize, i) the neurophysiological biomarkers of FoG, ii) the present implementations of aDBS, and iii) the remaining gaps that need to be addressed to unlock the full potential of aDBS in FoG.

## Markers from neuronal time series associated with the gait cycle and freezing of gait in Parkinson’s disease

Specific to the implementation of aDBS for FoG, neurophysiological biomarkers derived from neuronal activity measures like local field potentials (LFP) [50–53] or from kinematic recordings detected by wearable devices [54, 55] should identify the present motor state with high temporal resolution [51, 56–61]. Below, we will focus on oscillatory biomarkers from neuronal time series associated with successful walking and FoG in PD [54].

### Subthalamic nucleus activity is modulated during the regular gait cycle

Pathologically elevated oscillatory activity in STN-LFPs has long been associated with PD. In particular, low beta band activity (13–20 Hz) has been correlated with akinesia and rigidity [51, 52, 62, 63]. Furthermore, suppression of this exaggerated activity with levodopa or STN-DBS has been associated with clinical improvement [51, 64, 65]. More recently, several studies have started to investigate the spectral characteristics of STN activity and their relation to gait. These studies have indicated that beta band activity in PD patients is attenuated during unconstrained walking compared to standing [66] or sitting [35, 67], which is similar to the characteristics seen in cortical recordings from healthy subjects [68]. Besides, cyclical modulation of beta band activity has been shown to be time-locked to the sequence of repetitive leg movements of stepping-in-place (SIP) [69, 70], walking [35, 66, 71–73] and cycling [74].

More recent work has emphasized that the STN is involved in encoding the initiation and termination of gait, as well as the amplitude of leg muscle activation while walking across different walking states [75]. Specifically, low and high beta band activity have shown different levels of desynchronization at distinct phases of the gait cycle. Therefore, low and high beta band activity may represent distinct networks for the encoding of leg muscle synergies [76] given that these frequency bands are also modulated differentially in response to either dopaminergic medication or STN-DBS [61]. While levodopa primarily attenuates lower beta frequencies, the effects of STN-DBS also extend to higher beta frequencies [61]. Based on upper-limb movement tasks, the combination of both treatments has shown additional effects compared to each treatment alone [61, 77]. However, whether the dopaminergic state plays a role in gait-induced beta suppression or gait-cycle-related spectral perturbations has not been reported to date.

Further progress in characterizing the neurophysiological signals associated with FoG has been made in the past few years. Both the activity increase and the temporal dynamics of lower and higher beta activity have been associated with gait impairment in PD [78, 79] and differ significantly between freezers and non-freezers [74]. In line with previous reports [78], PD patients with reported FoG displayed increased activity around 15–21 Hz that showed particular enhancement at movement onset, unlike non-freezers [74, 80]. Together, this data suggests that patients experiencing FoG have disturbed cyclical STN beta modulation during gait.

### STN oscillatory activity of defective locomotor circuits and their relation to freezing of gait

A growing number of neurophysiological studies have reported oscillatory abnormalities of the STN and cortical activity that are associated with the freezing phenomena. Studies of upper-limb freezing have been conducted as hypothesis-generating work for FoG and point towards several activation abnormalities at the cortical level including increased alpha band activity and cortico-muscular coherence during freezing [81]. Furthermore, failure of event-related alpha and beta band spectral perturbation was present during the repetition cycle of regular finger tapping but absent during freezing and the immediate transition period preceding the freeze [82]. In recent years, the advent of ambulatory neurophysiological recording techniques including STN-LFP sensing and ambulatory EEG has produced a small number of studies investigating “true” FoG during overground walking which could not be achieved in traditional neuroimaging studies [83]. A summary of these selected studies reporting the neuronal activity during regular gait and FoG can be found in Table 1.

**Table 1:** Selected studies on the neuronal activity assessed during different motor states and Freezing of Gait in Parkinson’s disease patients

Feature	Study	Patient cohorts	Size (*n*)	Locomotor state	Target	Domain	Main findings
Low beta (13–20 Hz)	Singh et al. 2013	Freezers vs. non-freezers	6	Walking	STN	FD	Enhanced activity in freezers compared to non-freezers
	Chen et al. 2019	Advanced PD patients	15	Walking	STN	FD	Enhanced activity found at times of vulnerable gait susceptible to FoG
	Thenaisie et al. 2022	Advanced PD patients	20	Walking	STN	FD/TF	Enhanced activity during freezing compared to standing or walking
High beta (20–35 Hz)	Toledo et al. 2014	Freezers vs. non-freezers	22	Sitting	STN	FD	Enhanced activity in freezers compared to non-freezers
	Hell et al. 2018	Advanced PD patients	10	Walking	STN	FD/TF	Attenuation during walking with modulation across the gait cycle
	Fischer et al. 2018	Advanced PD patients	16	SIP	STN	FD/TF	Alternating modulation between left and right STN across the step cycle
Full beta (13–30 Hz)	Quinn et al. 2015	Advanced PD patients	15	Walking	STN	FD	Proportionally greater attenuation of power during walking compared to rest in AR vs. TD subjects (non-significant)
	Syrkin-Nikolau et al. 2017	Freezers vs. non-freezers	14	SIP	STN	FD/TF	Greater attenuation in freezers vs. non-freezers during SIP compared to rest
	Storzer et al. 2017	Freezers vs. non-freezers	13	Walking/ Bicycling	STN	FD/TF	Attenuation in both walking and bicycling compared to rest ~ 18 Hz oscillatory increase during and outside of FoG in freezers
	Anidi et al. 2018	Freezers vs. non-freezers	12	Walking/SIP	STN	FD/TF	Prolonged burst durations differentiated freezers from non-freezers Prolonged burst durations during FoG compared to walking
	Georgiades et al. 2018	Advanced PD patients	8	Walking^*‡^	STN	TF	Increased STN firing rate during FoG; Increase of beta and theta activity at beginning and throughout FoG episode
	Canessa et al. 2020	Advanced PD patients	8	Walking	STN	FD	Task-specific peak frequency modulation
	Louie et al. 2022	Advanced PD patients	3	Walking	STN	FD/TF	Gait-cycle specific modulation of theta, alpha, beta and low gamma power
	Klocke et al. 2024	Advanced PD patients	12	Walking	STN	FD/TF	Activation-deactivation abnormalities both before and during FoG Defective activation pattern of subthalamo-spinal circuits during FoG
Others	Pozzi et al. 2019	Advanced PD patients	7	Walking	M1-STN	FD/TF	M1-STN synchrony of theta-alpha range during walking; Increased M1-STN coupling of beta range and decoupling of theta-alpha range during FoG compared to walking
	Gulberti et al., 2023	Advanced PD patients	12	SIP^*^	ZI/STN/SN	–	STN firing rate changes in relation to attentional/motor aspects of SIP task Non-significant increase of irregular burst firing and increased beta activity during irregular stepping compared to regular stepping
	Choi et al. 2024	Advanced PD patients	18	Walking	STN	–	Deep-learning-based regression model predicted gait performance based on STN-derived features (8–100 Hz) rather than beta power alone (13–36 Hz)

Studies are given in chronological order of publication. *AR* akinetic rigid; *FD* frequency domain; *FoG* freezing of gait; Full beta- ~ 13 to ~ 30 Hz; High beta-~ 20 to ~ 30 Hz; *LFP* local field potential, Low beta- ~ 13 to ~ 20 Hz; *M1* motor cortex; *PD* Parkinson’s disease, *SIP* stepping in place, *SN* substantia nigra, *STN* subthalamic nucleus, *TD* tremor dominant, *TF* time frequency domain, *ZI* zona incerta; *intraoperative; ^‡^virtual reality task

Frequency domain analysis has revealed variable findings on beta band activity during FoG when compared to different reference conditions like walking, standing or sitting. In particular, activity was found to be increased across the entire beta band in FoG compared to sitting [78] or limited to the low beta band when compared to standing [75]. However, other studies using comparable [66] or different reference conditions in walking [69, 71] could not reproduce increased beta band activity during FoG. Heterogeneity across studies may stem from low statistical power due to small sample sizes ranging from 3 to 8 FoG patients, differences in experimental paradigms, and the variability in LFP normalization procedures. Time–frequency analyses have enabled closer insights into the temporal STN modulation time-locked to the onset of FoG. One such study using an intraoperative virtual reality gait paradigm found marked elevations of theta, as well as low and high beta band activity during FoG compared to baseline walking activity [84]. Another study demonstrated a numeric (non-significant) increase of beta band activity compared to standing [66]. Across studies, a common finding is that the cyclical pattern of event-related synchronization (ERS) and desynchronization (ERD), as seen in regular walking, is lost when patients experience FoG events which may as well extend to the immediate Pre-FoG period [85]. Of note, these altered STN activation patterns correlated closely with abnormal lower limb muscular dynamics (recorded from EMG), suggesting a pathophysiological link between deranged basal ganglia rhythmicity and FoG [66, 75, 84].

More granular work on STN beta activity has identified short-lived phasic bursts representing physiological signal processing in the sensorimotor network [52, 86]. In particular, long-duration bursts occur predominantly in the Parkinsonian off-state and can be correlated with motor impairment [52] and specifically FoG episodes [87]. Furthermore, long-duration bursts were shown to differ between freezers and non-freezers at rest and were increased at the onset of FoG compared to regular gait [87]. Finally, STN beta modulation during FoG differed from that observed during walking or with volitional stops [66, 84]. At the cortical level, it has been suggested that the sensorimotor, frontal, parietal and occipital areas are involved in both occurrence and compensation of FoG [88]. The primary motor cortex (M1) related to the control of gait initiation and gait stability [89, 90] is also involved in gait impairments and FoG. To this end, effective compensation strategies like acoustic cueing can lead to a decrease of beta band activity in these sensorimotor areas [88]. Similarly, it has been reported that internal cueing can lead to a change in frontal activation, while external cueing can decrease parieto-occipital alpha activation. Besides, subthalamo-cortical decoupling of alpha- and theta band synchronization has been found prior to and during FoG that resolved upon the reinstatement of regular gait [71]. Invasive electrocorticography (ECoG) recordings can assess beta-gamma phase-amplitude coupling (PAC) in the motor cortex of PD subjects [91]. It has long been proposed that PAC may represent a mechanism for communication within and across different brain regions by coordinating the timing of neuronal activity in brain networks [92]. Significantly, freezing episodes have been associated with a higher beta-gamma PAC compared to normal walking [91, 93] and it should also be highlighted that STN-DBS can alleviate FoG in parallel with decoupling of beta-gamma PAC [91].

### Markers of “network instability” that indicate an increased freezing susceptibility

The transition period between effective stepping and freezing represents a potential “window of opportunity” for effective interventions to abort these episodes. Therefore, biomarkers representing this emerging “network instability” are of significant interest and may be indicative of increased freezing susceptibility. Indeed, findings from upper-limb freezing have supported this view where increases of cortico-cortical beta band synchronization, as well as diminished beta band modulation, have been recorded during such transition periods and were associated with increased freezing susceptibility [82, 94].

Similarly, there is a susceptibility toward FoG as illustrated by the tendency for it to be triggered by circumstances that require adjustment of the locomotor program like gait initiation, turning, approaching destinations or bypassing obstacles. Thus, “computational overload”, as postulated in the neural reserve hypothesis or cognitive interference model, may drive an unstable locomotor system to the point at which there is an overload of the compensatory capacities of the network, resulting in FoG [95, 96]. Neurophysiological markers have added support to this framework and may reflect brain states of increased FoG susceptibility at various nodes of the locomotor system. For example, at the STN level, gait cycle-related modulations of beta band activity are attenuated not only during the freeze, but up to three steps before it [66]. Furthermore, cognitive interference while walking, known as being one of the strongest provoking maneuvers to elicit FoG, attenuated the gait-cycle-related beta band modulations in the same work. Interestingly, the STN was synchronized to the defective activation and timing pattern of the spinal motor neurons, and this was evident both before freezing, and when contrasting “freezers” that did versus those patients that did not show FoG during the experimental session [66]. Similarly, increased lower beta band activity from 15 to 21 Hz alongside increased theta activity was found in other work when depicting “gait vulnerable to freezing” from a kinematic vulnerability index [80] and similar findings have also been reported by ambulatory EEG [85, 97, 98]. This and other work suggested that theta band activity might reflect increased freezing susceptibility [71, 84, 98] albeit this has not been reproduced by all LFP studies [66, 75].

Theta rhythms may act as a mechanism of long-range synchronization between the STN and the frontal cortices and are relevant and sensitive to the cognitive context and executive control of gait, which might therefore show variable expression across studies [71, 99–101]. Other nodes of the locomotor system include the GPi and the SNr, but no recordings of oscillatory features from these structures have been reported in relation to gait. Only one SNr study based on intraoperative microelectrode recordings has suggested that SNr neurons are more sensitive to cognitive as opposed to motor contents of an intraoperative stepping task in the supine position[102]. In another study, theta oscillations localized at the ventral STN–SNr border zone pointed to increased FoG vulnerability [80]. Very few neurophysiological studies have been undertaken in patients with pedunculopontine electrodes, but those that have suggested that alpha band activity is correlated with gait performance and suppression of alpha band activity one second before and during FoG [103, 104].

## Present technical implementation of adaptive DBS

To date, aDBS has not been specifically utilized for the treatment of FoG. However, accurately characterizing the neuronal oscillations associated with the dopaminergic off-state has built a strong framework for existing closed-loop devices to be clinically applied. Indeed, a number of studies have demonstrated that aDBS can offer equivalent or even greater symptomatic relief than cDBS for symptoms of bradykinesia, rigidity and dyskinesia [37–42, 105–107]. Furthermore, these works have highlighted that aDBS may offer advantages in efficiency by lowering the total energy delivered [108] which would potentially reduce stimulation-induced side effects such as dysarthria [40]. Outcomes of significantly improved on-time compared to cDBS have also been reported with aDBS where alpha–beta LFP power (8–30 Hz) has been used as a feedback signal [45, 54]. More recently, preliminary insights from the largest prospective multicenter trial of aDBS to date in patients with PD have been released (ADAPT-PD Trial – NCT04547712) [54]. As outlined below, this work has suggested that a range of technical implementations will need careful consideration for the specific use of aDBS in the treatment of FoG.

### Stimulation control based on thresholding techniques

Programming aDBS based on a single LFP threshold has been widely used in clinical studies [37, 44, 47, 48, 54, 105, 109, 110], where the algorithm is designed to suppress beta activity over short time intervals (a few hundreds of milliseconds), once the amplitude threshold is exceeded. The threshold amplitude is derived from the most prominent spectral activity peak within the alpha–beta frequency range of an individual patient based on subjective clinician judgment and is estimated every 100 ms epoch. Elapse of the “onset time” will lead to an adjustment of the stimulation amplitude, which is mostly calibrated within a range of 200–500 ms with a rapid ramping time of ~ 250 ms. This means that the single-threshold control policy is in principle able to react on a short time scale to changes in the time–frequency spectrum. As such, it enables the desynchronization of long-duration beta bursts, which typically indicate the clinical off-state [52]. Since FoG has also demonstrated an increase in the long-duration burst around the onset of a freeze, it would be worth studying this single-threshold approach further, if such an adaptive control algorithm were to have lasting effects on FoG[87]. Indeed, provided that STN-DBS is able to suppress these long beta bursts, it may have benefits for levodopa-sensitive FoG.

Alternatively, the proposed dual-threshold control policy follows a different conceptualization by adjusting stimulation amplitudes over longer time intervals, sequentially over minutes rather than with a sharp ramp within a few hundreds of milliseconds. This concept is intended to tailor stimulation to the medication dose cycle in order to stabilize dopaminergic fluctuations [54]. Currently, this dual-threshold approach has been used to treat FoG only in a single patient [46]. While such adaptive stimulation control might be too tardy to react to single FoG episodes, it offers a more personalized medicine approach for patients experiencing predominantly FoG related to the dopaminergic off-state.

### Experimental custom-made control policies for freezing of gait

Currently, within the research setting, there are a number of custom-made algorithms that are being evaluated, such as placing a “PC-in-the-loop” using the Summit™ PC + S impulse generator from Medtronic (Minneapolis, MN, USA) [48, 110] (Table 2). These studies have used an interconnected system that enables the collection of continuous data-sensing via telemetry and an external host computer to update the stimulation parameters in real-time [111]. The first study to adapt DBS based on gait kinematics from inertial measurement units (APDM Inc.) was conducted in a single patient while walking to predict the freezing probability based on an earlier validation study [107, 112]. FoG classification was based on gait parameters including leg arrhythmicity, stride time, and leg asymmetry over the last six steps which was used to control both stimulation amplitudes and frequencies (60 vs. 140 Hz) upon crossing pre-set FoG probability thresholds. Despite being a benchtop-validation study, the results showed technical feasibility and will have to be tested in affected patients next. More recent work from the same group has employed a beta burst-driven approach, where beta bursts were first computed in a patient-specific 6 Hz range within the beta band range (13–30 Hz) with changes in stimulation being triggered using a single-threshold control policy [48]. This approach reduced the %-time spent with freezing compared to when the stimulation was turned off and had a similar efficacy compared to continuous DBS. Ongoing work is seeking to address whether aDBS exerts its benefits through temporal alignment of these long beta bursts and if this approach will truly outperform continuous DBS (NCT04043403).

**Table 2:** Current clinical and feasibility studies of adaptive deep brain stimulation in Parkinson’s disease with focus on freezing of gait

Study	Samples (*n*)	Stimulation target	Output signals	Main results
O’Day et al. 2020	1 healthy subject 1 PD patient with FoG (2)	Unimplanted DBS-device‡﻿	IMU	▪ Setting*:* free walking (self-control) ▪ Stimulation time: no information available ▪ Adaptive strategy: single-threshold, dual threshold (Nexus D) ▪ Algorithms: gait arrhythmicity and logistic regression model used as threshold parameters ▪ Results: novel control policy algorithm to change frequency or stimulation intensity in response to kinematic inputs; No clinical outcomes reported due to the use of pre-recorded kinematic data
Petrucci et al. 2020	PD patient with FoG (1)	Bilateral STN	STN-LFP	▪ Setting: SIP (self-control) ▪ Stimulation time: 2 min ▪ Adaptive strategy: gradual, dual-threshold control determined from beta band power during movement (± 3 Hz around peak frequency of elevated beta band power during SIP) ▪ Results: threshold set at average beta power during SIP task measured at min. and max. DBS voltages that showed improvement during SIP task; aDBS was superior in reducing FoG (%-time freezing: 67.7% OFF DBS, 2.3% cDBS, 1.5% aDBS); SIP arrhythmicity was lower in aDBS
Molina et al. 2021	PD patients with medication-refractory FoG (5)	Bilateral GPi^*^ Bilateral PPN	Unilateral PPN LFP	▪ Setting: in-laboratory and outside hospital (Self-control) ▪ Stimulation time: 5–15 months ▪ Adaptive strategy: single-threshold (Nexus D/E) with ON/OFF PPN stimulation as power within PPN (1–8 Hz band) crosses patient-specific threshold determined from prior off-stimulation period; patient-specific threshold determined based on performance classification (ROC) and stimulation duration (3.5 s) determined from maximized ROC AUC ▪ Results: 40% device removal rate due to infection, 14 related AE, 7 severe AE; > 40% improvement in FoG in 60% of subjects at 6 months using aDBS; Heterogenous clinical effect in FoG outcomes (non-significant with cDBS)
Wilkins et al. 2024	Advanced PD patients with FoG (7)	Bilateral STN	STN-LFP	▪ Setting: testing in medication off-state, free walking (Self-control) ▪ Stimulation time: 120 min ▪ Adaptive strategy: single-threshold (Nexus D), based on subject-specific burst durations threshold ▪ Results: overall %-time freezing and mean peak shank angular velocity improved from OFF to aDBS; aDBS showed similar efficacy as cDBS on FoG, tremor, bradykinesia and rigidity

*aDBS* adaptive deep brain stimulation, *AE* adverse event, *AUC* area under the curve, *cDBS* conventional deep brain stimulation, *FoG* freezing of gait, *IMU* inertial measurement unit, *PD* Parkinson’s disease, *PPN* pedunculopontine nucleus, *ROC* receiver operating curve, *SIP* stepping in place, *STN* subthalamic nucleus, ^‡^This feasibility study used both real-time human kinematic data and kinematic data previously recorded from a PD patient. The closed-loop system was, however, not yet tested in a human subject. ^*^Bilateral GPi was used for open-loop deep brain stimulation to address levodopa-responsive PD symptoms while both PPN were the target of aDBS

## A roadmap to develop aDBS for freezing of gait

Implementing adaptive DBS to treat FoG appears promising but currently faces both methodological and technical boundaries that need to be overcome. Building on the difficulty to identify and select a robust control signal for FoG, we suggest exploring several key areas.

### The heterogenous phenomenology of freezing of gait

It is unlikely that targeting a single FoG biomarker will be possible given its heterogeneous clinical presentations (e.g., start hesitation, destination freezing, freezing when passing narrow obstacles, freezing when turning, trembling-in-place-like versus akinetic freezing). Furthermore, it is likely that non-motor cognitive and emotional contexts will significantly modulate the susceptibility and occurrence of FoG in a bidirectional manner. This complexity has not yet been studied in terms of the involved neuronal basal-ganglia circuits, transmitters and their correlates in terms of oscillatory biomarkers. Presumably, subthalamic pathological beta band activity may play a role before and during a freeze across these situations, but it should be expected that several basal ganglia–cortical circuits are involved within a complex interplay which needs pathophysiological differentiation. Further, cDBS has primarily proven effective for levodopa-sensitive FoG and it can be anticipated that aDBS tailored to beta band characteristics (spectral amplitude, bursts) will do so similarly. However, how to address levodopa-resistant FoG would be among the most meaningful steps forward, yet has not been achieved at all with any type of control signal or stimulation.

**Fig. 1:**
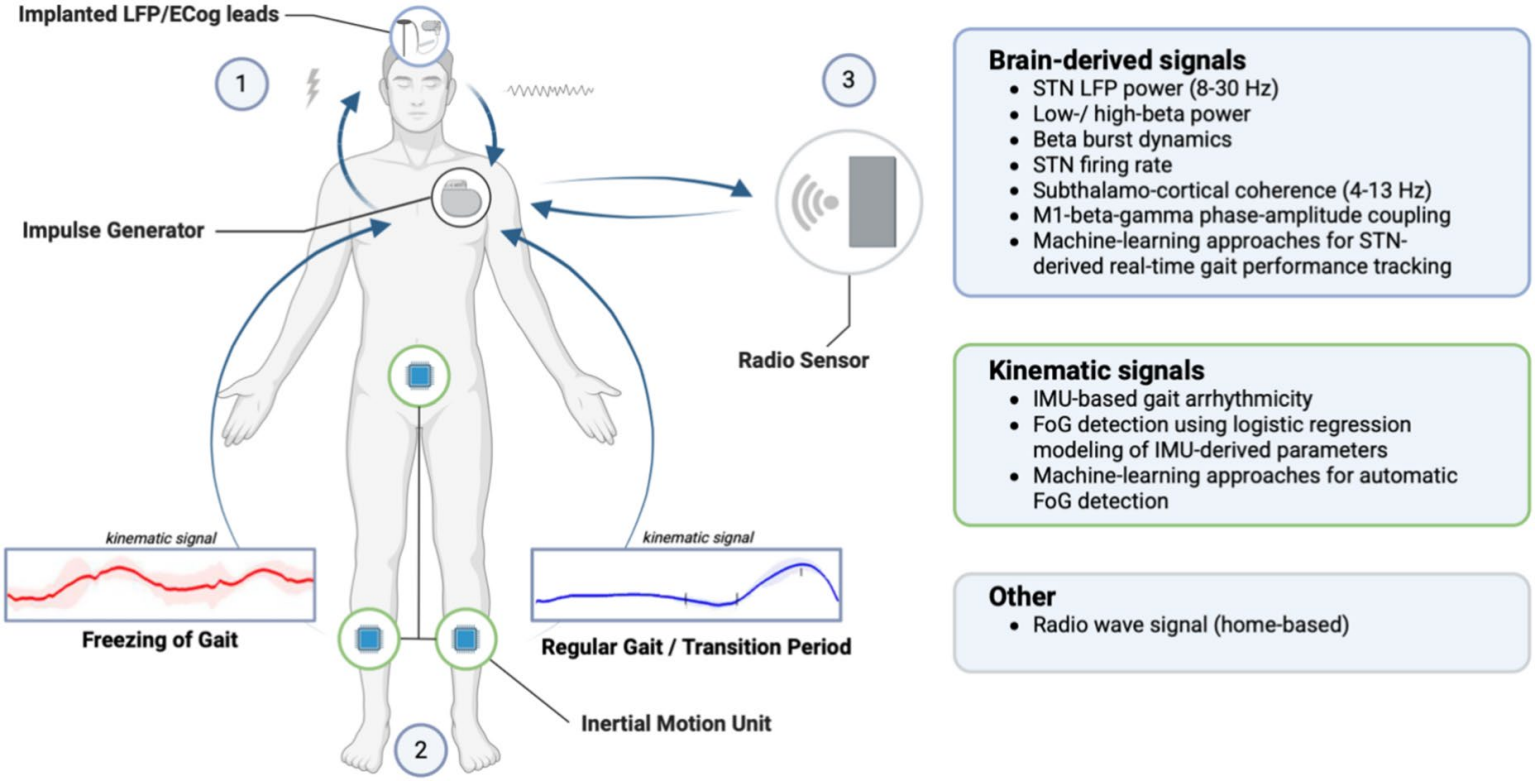
Schematic representation of different input markers for potential implementation of adaptive deep brain stimulation (DBS) in the treatment of freezing of gait (FoG). (1) Brain-derived signals from either implanted subthalamic DBS electrodes or electrocorticography (ECog) leads are used to inform the impulse generator of a state susceptible to FoG which results in a closed-loop adaptation of the stimulation parameters. (2) Inertial measurement units (IMU) attached to both shanks and/or lumbar vertebrae are used to track gait in real-time and to detect FoG and its preceding transition period which is transmitted to the impulse generator. (3) Gait is monitored using radio waves with a device set up at a patient’s home to extract walking movements and trajectories through the home. In theory, all input markers can be used individually or in combination to best reflect a patient’s freezing phenomenology

Another challenge is replicating laboratory observations in a home-based (unsupervised) setting. Albeit advances have been made with wearable technology to extract FoG from inertial measurement unit time series with machine learning, the diagnostic accuracy has repeatedly been found to be still limited in home-based settings [55]. Recent studies utilizing radio signals have suggested this possibility as an alternative approach, but this has yet to be adapted to FoG [113]. A multitude of factors including fluctuating dopamine levels [106, 114], circadian rhythms [115, 116], mobility state [66], and potential signal artefacts [117, 118] may also modulate oscillatory biomarkers, further complicating their interpretation and potentially limiting classification accuracy. A summary figure illustrating the interplay of different biomarkers used individually or in combination for potential implementation in aDBS is given in Fig. 1.

### Signal processing – the challenge of developing robust algorithms

LFPs combine a complex mix of oscillatory and non-oscillatory neural activity that may provide insights beyond beta power alone, including a diverse set of metrics like activity, synchronization, phase-amplitude coupling, burst dynamics, and many more. Indeed, this activity is likely to reflect many different neuronal mechanisms and circuit processes occurring at differing degrees over time, such as executive function [119] and neuropsychiatric symptoms [120], rather than being specific to FoG [59, 121–124]. This high degree of dimensionality will require algorithms that extend from simple linear thresholding techniques of a single marker (like beta band activity) to the integration of more complex classification routines involving a set of markers and non-linear classification. For this, machine learning may represent a powerful tool to support classification personalized to an individual patient with high temporal resolution [125]. This would provide the opportunity for a non-biased classification routine and real-time adjustments that could potentially yield advantages over clinician-based programming [126].

When conceptualizing effective adaptive neuromodulation for FoG, it may be more effective to focus on the “facilitation of preserved gait” (i.e., lowering the susceptibility to a freeze), as opposed to aborting individual FoG events. To this end, a small number of studies have started to explore the neuronal correlates of freezing susceptibility [66, 70, 80, 82, 94]. This conceptual approach would make clinical sense to reduce the occurrence of freezing by stabilizing gait performance. It would also align better with neurostimulator control, providing a larger time window to detect and stabilize the spectral perturbations associated with the gait cycle. In this framework, an input from simultaneous gait kinematics that adapt stimulation to the gait cycle would also be of interest. While such data cannot be currently fed into available neurostimulator devices, this could be a focus of future research. Clinicians, researchers, engineers, and industry partners will need to work in close liaison to overcome the present technical constraints to make aDBS widely available for FoG.

### What might future clinical trials look like?

Obviously, any intervention would need to be validated in a clinical trial setting and there is a current issue with standardizing endpoint measures with a move away from self-reported questionnaires [127], as well as the ability for home-based recordings [55]. Future trial design would also have to account for continuous versus adaptive interventions [37, 54]. Another problem with FoG trials involves the endpoint definition: scales like the FOG-Q / NFOG-Q are based on a patient's self-reporting, which may be subject to bias and even more difficult to interpret once patients develop cognitive impairment as a common co-incidence with FoG. In contrast, specific FoG parkours provide validated and objective outcome measures on FoG although they fail to capture a patient’s state in their natural ecological setting. Wearable technology was seen as a possibility to close this gap, but albeit significant progress has been made, classification accuracy is still limited in the presence of artificial intelligence [55]. Finally, validated ecological outcomes that impact on the daily activities and quality of life of patients will need to become established [8, 128].

## Concluding remarks

With the advent of brain sensing-enabled devices, the field is gaining more mechanistic insights into the complex architecture that defines FoG in PD. However, conceptualization of aDBS for FoG is lacking a differentiated understanding of the appropriate oscillatory biomarker. The heterogeneity of FoG, as well as its modulation within the context of cognition and emotional state make this symptom difficult to capture with a single oscillatory marker. Nevertheless, preliminary insights exist to characterize and identify locomotor network vulnerability in terms of an increased likelihood of FoG expression. This may help conceptualize aDBS applications that help stabilize brain oscillations relating to the regular locomotor rhythms, thereby lowering the risk to progress to FoG events. To achieve this, technological innovations are needed, moving forward from the present linear threshold control to more sophisticated neuronal interfaces that allow the implementation of multimodal biomarkers and enhanced classification routines. Close cooperation between clinicians, researchers, engineers, and industry will be needed to achieve these aims.

## Data Availability

Data availability is not applicable to this article as no new data were created or analysed in this study.
